# Molecular transmission network analysis of newly diagnosed HIV-1 infections in Nanjing from 2019 to 2021

**DOI:** 10.1186/s12879-024-09337-6

**Published:** 2024-06-12

**Authors:** Hongjie Shi, Xin Li, Sainan Wang, Xiaoxiao Dong, Mengkai Qiao, Sushu Wu, Rong Wu, Xin Yuan, Jingwen Wang, Yuanyuan Xu, Zhengping Zhu

**Affiliations:** 1 Department of AIDS/STD Control and Prevention, Nanjing Center for Disease Control and Prevention, Nanjing, China; 2 Department of Laboratory Medicine, Jiangning Hospital Affiliated to Nanjing Medical University, Nanjing, China; 3 Department of Microbiology Laboratory, Nanjing Center for Disease Control and Prevention, Nanjing, China

**Keywords:** HIV, Molecular transmission network, Clusters, Transmitted drug resistance

## Abstract

**Objective:**

The objective of this study was to conduct a comprehensive analysis of the molecular transmission networks and transmitted drug resistance (TDR) patterns among individuals newly diagnosed with HIV-1 in Nanjing.

**Methods:**

Plasma samples were collected from newly diagnosed HIV patients in Nanjing between 2019 and 2021. The HIV *pol* gene was amplified, and the resulting sequences were utilized for determining TDR, identifying viral subtypes, and constructing molecular transmission network. Logistic regression analyses were employed to investigate the epidemiological characteristics associated with molecular transmission clusters.

**Results:**

A total of 1161 HIV *pol* sequences were successfully extracted from newly diagnosed individuals, each accompanied by reliable epidemiologic information. The analysis revealed the presence of multiple HIV-1 subtypes, with CRF 07_BC (40.57%) and CRF01_AE (38.42%) being the most prevalent. Additionally, six other subtypes and unique recombinant forms (URFs) were identified. The prevalence of TDR among the newly diagnosed cases was 7.84% during the study period. Employing a genetic distance threshold of 1.50%, the construction of the molecular transmission network resulted in the identification of 137 clusters, encompassing 613 nodes, which accounted for approximately 52.80% of the cases. Multivariate analysis indicated that individuals within these clusters were more likely to be aged ≥ 60, unemployed, baseline CD4 cell count ≥ 200 cells/mm^3^, and infected with the CRF119_0107 (*P* < 0.05). Furthermore, the analysis of larger clusters revealed that individuals aged ≥ 60, peasants, those without TDR, and individuals infected with the CRF119_0107 were more likely to be part of these clusters.

**Conclusions:**

This study revealed the high risk of local HIV transmission and high TDR prevalence in Nanjing, especially the rapid spread of CRF119_0107. It is crucial to implement targeted interventions for the molecular transmission clusters identified in this study to effectively control the HIV epidemic.

**Supplementary Information:**

The online version contains supplementary material available at 10.1186/s12879-024-09337-6.

## Background

Since the first case of AIDS was reported in China in 1985 [1], nearly 40 years have passed. In 2018, the Chinese government estimated that there were approximately 1,250,000 people living with HIV/AIDS in the country [2]. Despite the implementation of various strategies, such as promoting condom usage among female sex workers and men who have sex with men (MSM), providing syringe service programs for injecting drug users, offering mother and child block interventions for HIV-positive pregnant women, as well as making pre-exposure prophylaxis (PrEP) and post-exposure prophylaxis (PEP) available to all populations, the number of newly reported HIV infections per year has unfortunately not shown a significant decline. This situation is a cause for concern and underscores the persistent challenges faced in HIV prevention and control efforts in China.

In 2018, the US Centers for Disease Control and Prevention (CDC) proposed the use of HIV molecular transmission networks as a new strategy for HIV prevention [3]. This approach has gained recognition and was included as one of the main strategies in the US Department of Health and Human Services' ambitious plan "Ending the HIV Epidemic: A Plan for America" in 2019 [4]. In line with these developments, the National Center for AIDS/STD Control and Prevention at the Chinese Center for Disease Control and Prevention published guidelines for monitoring and intervening in HIV transmission networks in China (trial version) in September 2019 [5]. HIV molecular transmission networks are constructed using genetic data from individuals infected with HIV. By identifying similarities and connections between viral sequences, these networks aim to reconstruct the macro-social networks of infected individuals and examine the characteristics of the network's active and critical groups, with the ultimate goal of preventing and controlling HIV transmission [6]. Over the years, research on molecular transmission networks has expanded, not only helping analyze the basic characteristics of the epidemic but also playing a role in determining when and where newly diagnosed individuals were likely infected, assessing the speed of HIV transmission spread, and guiding intervention efforts.

The use of HIV molecular transmission networks has shown promise in understanding the dynamics of HIV transmission and designing targeted prevention strategies. By identifying clusters of interconnected infections, public health officials can prioritize interventions and resources to effectively reach populations at higher risk. This approach allows for a more precise understanding of transmission patterns and can inform the development of tailored prevention and control measures.

In China, the adoption of HIV molecular transmission networks as a strategy reflects the country's commitment to staying at the forefront of HIV prevention and control efforts. By leveraging genetic data and analyzing transmission networks, China aims to enhance its understanding of the epidemic, identify key populations, and implement interventions that can have a significant impact on reducing new HIV infections [7].

Continued research and collaboration are necessary to refine the use of HIV molecular transmission networks in China. This includes strengthening laboratory capacities for genetic sequencing, improving data sharing and integration, and ensuring the ethical use of this information while protecting individuals' privacy. By harnessing the potential of molecular transmission networks, China can further enhance its HIV prevention and control efforts and work towards reducing the burden of HIV/AIDS in the country.

In China, the predominant HIV-1 subtypes are CRF01_AE and CRF07_BC [8], as well as some second-generation recombinant forms such as CRF01_AE/CRF07_BC, CRF01_AE/CRF08_BC, CRF07_BC/CRF55_01B and so on have been reported to be prevalent in different regions and different high-risk populations. Different HIV-1 subtypes, different high-risk groups and different regional distribution result in different HIV transmission characteristics, which also poses challenges to HIV epidemic prevention and control. Nanjing, as the capital city of Jiangsu Province and one of the central cities of the Yangtze River Delta Economic Belt, boasts a highly developed cultural tourism and transportation network. The unique geographical characteristics of Nanjing contribute to its distinct epidemic characteristics. Currently, the predominant mode of HIV transmission in Nanjing is through homosexual contact, accounting for 69.0% of cases, which is higher than the provincial and national averages [9–11]. The frequent population movement driven by social and economic development further complicates the HIV epidemic in Nanjing, leading to diverse and complex transmission patterns. Given the complexity of the epidemic in Nanjing, it is crucial to understand the distribution characteristics of HIV subtypes and identify transmission clusters using molecular transmission networks. This approach provides valuable insights into the local HIV epidemic strains, their subtypes, and the interconnectedness of infections. By analyzing transmission networks, public health officials can formulate targeted intervention strategies and allocate resources more effectively. This data-driven approach helps prioritize interventions and implement measures that can have a significant impact on reducing new HIV infections in Nanjing.

## Material and methods

### Study population and sample collection

This study included all newly diagnosed HIV-1 patients in Nanjing from January 2019 to December 2021 who had not received antiretroviral therapy (ART). Informed consent was obtained from each participant before their inclusion in the study. A peripheral blood sample of 6 mL was collected from each participant within 12 h of collection and stored at -80 °C for further analysis. The baseline CD4^+^ T lymphocyte (CD4) cell count and HIV viral load (VL) data were collected from patients who were diagnosed before starting ART. Additionally, epidemiological information including age, sex, and transmission route of infection was surveyed at the time of enrollment. This study was conducted in accordance with the ethical guidelines and was reviewed and approved by the ethics committees of Nanjing Center for Disease Prevention and Control.

### Laboratory operations

The extraction of viral RNA was performed using the QIAamp Viral RNA Mini Kit (Qiagen, Hilden, Germany) following the manufacturer's protocol, with 200 μl plasma samples used for each extraction. Nested polymerase chain reaction (PCR) was used to amplify the target fragment containing 1060 bp in the *pol* gene (HXB2:2253–3313). The first-round PCR procedure and cDNA synthesis were carried out using PrimeScriptTM One Step RT-PCR Ver. 2.0 (TakaRa, China). Cycling conditions were 50 ℃ for 45 min; 94 ℃ for 2 min; 94 ℃ for 15 s; 55 ℃ for 20 s; 72 ℃ for 2 min, 50 cycles; followed with an extension at 72 ℃ for 10 min. The nested PCR was conducted using Ex Taq (TaKaRa, China). Cycling conditions were 94 °C for 4 min; 94 °C for 15 s, 55 °C for 20 s, 72 °C 2 min, 40 cycles; followed with an extension at 72 °C for 10 min. PCR products were validated by visualizing 1% agarose gel electrophoresis results. Successfully amplified samples were sent to Sangon Biotechnology Co. for sequencing using Applied Biosystems 3730XL. The primers for PCR and sequencing are listed in Additional Table S1.

### HIV sequence acquirement and subtyping

Sequencher 4.10.1 (Gene Codes Corporation, Ann Arbor, MI, USA) was utilized for sequence splicing, and the aligned sequences were analyzed using BioEdit (version 7.0.9, Informer Technologies Inc.). To ensure sequence quality, the WHO HIVDR QC TOOL (Resistance Quality Control Tool provided by the World Health Organization, https://sequenceqc.bccfe.ca/who_qc) was employed. Sequences longer than 1000 bp and had less than 5% ambiguous nucleotides been included for analysis. FastTree 2.1, utilizing the maximum likelihood (ML) method, was used to generate a phylogenetic tree for subtype identification. The nucleotide substitution model GTR + G + I was applied, and support values of the nodes were calculated using a Shimodaira Hasegawa-like test. Clusters with a bootstrap value greater than 0.90 (90%) were classified as belonging to the same subtype. Reference sequences from the HIV Databases (https://www.hiv.lanl.gov/content/index) were used, which encompassed major international epidemic strains A-D, F–H, and J K, as well as the major epidemic recombinant strains found in China. The ML trees were visualized and edited using Figtree v1.4.3.

### TDR analysis and HIV molecular transmission network construction

The partial *pol* genes were aligned and uploaded to the Stanford HIV Drug Resistance Database website (https://hivdb.stanford.edu/) for TDR and mutations analysis [12]. The analysis of TDR categories involved protease inhibitors (PIs), nucleoside reverse transcriptase inhibitors (NRTIs) and non-nucleoside reverse transcriptase inhibitors (NNRTIs). Low-level resistance, intermediate resistance, and high-level resistance were identified as TDR among ART-naïve individuals [13]. Pairwise genetic distances were calculated using the Tamura-Nei 93 model, and HIV TRACE was employed to construct a molecular transmission network [14]. In the network, nodes represent individuals, while edges indicate two connected nodes with potential transmission relationships under a certain gene threshold. Links refer to the number of edges, also known as degrees, to which each node connects. The largest number of clusters could be found at the optimal gene distance threshold [15]. At this threshold, the network can identify the most transmission clusters. When the threshold increases more, different transmission clusters begin to gather with each other and the number of clusters decreases, suggesting the network's resolution ability decreases. Therefore, at a genetic threshold of ≤ 1.50%, individuals entering the cluster represented a potential transmission link. Small clusters contained ≤ 10 nodes, while large clusters contained more than 10 nodes in this study.

### Variable definition

The sample sources were categorized into three groups: VCT, hospital, and others. VCT refers to Voluntary Counseling and Testing conducted by CDC or Community Based Organizations (CBO) workers. Hospital samples included those from preoperative testing, sexually transmitted diseases (STDs) clinics, pre-blood (product) testing, testing of other patients, premarital medical examinations, as well as pregnancy and prenatal examinations. The "others" category included samples from physical examinations, blood donation tests, and the detection of compulsory/reeducation through labor drug rehabilitation personnel, among others. Education level was divided into two categories: low education, which included individuals with a middle school education or below, and high education, which included individuals with a senior high school education or above. For the route of transmission, individuals classified as injecting drug users (IDUs) or those with an unknown transmission route were categorized as "others."

### Statistical analysis

Statistical analysis was performed using R v4.1.3. Descriptive statistics, such as interquartile ranges (IQR) and medians, were used to summarize continuous variables with non-normal distributions. Categorical variables were described using frequencies and percentages. Univariate and multivariate logistic regression models were used to analyze the factors associated with transmission within molecular clusters or large molecular clusters. The variables with significance (*P* < 0.05) in the univariate analysis were included in the multivariate logistic regression model for analysis. All statistical tests were two-tailed, and a *p*-value of less than 0.05 was considered statistically significant.

## Results

### Basic characteristics and subtype distributions

A total of 1161 newly diagnosed HIV-positive individuals were included in our study from 2019 to 2021. Among them, 93.88% were male, and the median age was 29 years old (with an interquartile range [IQR] of 24–43). The majority of the participants (97.93%) belonged to the Han ethnicity, and 62.45% were unmarried. In terms of occupation, 48.15% were employees, 16.11% were students, 15.33% were unemployed, and the remaining 12.83% and 7.58% were classified as "others" and "peasants," respectively. Regarding education level, 77.95% of the participants had a high level of education. Approximately 52.54% of the samples were obtained from voluntary counseling and testing (VCT) centers, and 17.05% of the participants had already developed AIDS. Among these individuals, 77.09% had a baseline HIV viral load (VL) above 1000 copies/ml, while 56.42% had a CD4 cell count between 200 and 500 cell/mm^3^. Additionally, 17.31% had been previously diagnosed with sexually transmitted diseases (STDs), and 7.84% exhibited TDR.

In terms of transmission routes, 68.91% of the participants were infected through homosexual behaviors, followed by heterosexual behaviors (29.97%), and other behaviors (1.12%) (Table 1).

**Table 1:** Demographic and clinical patient characteristics associated with the probability of belonging to a molecular transmission cluster

Variables	Subjects *N* (%)^a^	Clustered *N* (%)^b^	Univariate analysis		Multivariate analysis	
			*OR*(95%*CI*)	*P*	*aOR*(95%*CI*)	*P*
Gender						
Male	1090(93.88)	569(52.20)				
Female	71(6.12)	44(61.97)	1.49(0.91–2.44)	0.112		
National						
Han	1137(97.93)	604(53.12)				
Others	24(2.07)	9(37.50)	0.53(0.23–1.22)	0.135		
Marital status						
Married	336(28.94)	190(56.55)				
Unmarried	725(62.45)	367(50.62)	0.79(0.61–1.02)	0.072		
Divorced	100(8.61)	56(56.00)	0.98(0.62–1.53)	0.923		
Age (years)						
16 ~ 30	629(54.18)	323(51.35)				
31 ~ 59	430(37.03)	219(50.93)	0.98(0.77–1.26)	0.893	1.00(0.74–1.34)	0.977
≥ 60	102(8.79)	71(69.61)	2.17(1.40–3.44)	< 0.001	2.10(1.18–3.79)	0.013
Occupation						
Unemployed	178(15.33)	103(57.87)				
Students	187(16.11)	92(49.20)	0.71(0.47–1.07)	0.098	0.63(0.40–1.00)	0.053
Employee	559(48.15)	276(49.37)	0.71(0.51–0.99)	0.049	0.67(0.47–0.96)	0.029
Peasant	88(7.58)	61(69.32)	1.65(0.96–2.83)	0.072	1.11(0.60–2.07)	0.752
Others	149(12.83)	81(54.36)	0.87(0.56–1.34)	0.525	0.67(0.41–1.09)	0.110
Education						
Low	256(22.05)	155(60.55)				
High	905(77.95)	458(50.61)	0.67(0.50–0.89)	0.005	0.76(0.53–1.09)	0.136
Sample source						
VCT	610(52.54)	297(48.69)				
Hospital	443(38.16)	248(55.98)	1.34(1.05–1.71)	0.019	1.20(0.91–1.60)	0.195
Others	108(9.30)	68(62.96)	1.79 (1.18–2.73)	0.007	1.69(1.09- 2.66)	0.021
Disease classification						
AIDS	198(17.05)	91(45.96)				
HIV	963(82.95)	522(54.21)	1.39(1.02–1.89)	0.035	0.92(0.54–1.55)	0.757
Baseline HIV VL (log RNA copies/ml)						
≤ 3	57(4.91)	27(47.37)				
> 3	1104(95.09)	586(53.08)	1.26(0.74–2.14)	0.405		
Baseline CD4 cell count (cell/mm^3^)						
< 200	260(22.39)	117(45.00)				
200 ~ 500	655(56.42)	362(55.27)	1.51(1.32–2.02)	0.005	1.95(1.20–3.21)	0.008
> 500	246(21.19)	134(54.47)	1.46(1.03–2.08)	0.034	2.03(1.19–3.50)	0.010
Transmission route						
Homosexual	800(68.91)	407(50.88)				
Heterosexual	348(29.97)	196(56.32)	1.25(0.97–1.61)	0.090		
Others^c^	13(1.12)	10(76.92)	3.22(0.88–11.78)	0.078		
STD history						
Yes	201(17.31)	103(51.24)				
No	810(69.77)	435(53.70)	1.10(0.81–1.50)	0.532		
Unknown	150(12.92)	75(50.00)	0.95(0.62–1.45)	0.818		
TDR						
Yes	91(7.84)	44(48.35)				
No	1070(92.16)	569(53.18)	1.21(0.79–1.86)	0.377		
Subtype						
CRF01_AE	446(38.41)	208(46.64)				
CRF07_BC	471(40.57)	250(53.08)	1.29(1.00–1.68)	0.051	1.17 (0.89–1.53)	0.255
CRF105_0107	73(6.29)	63(86.30)	7.21(3.61–14.41)	< 0.001	8.36(4.32–17.90)	< 0.001
CRF67_01B	37(3.19)	30(81.08)	4.90(2.11–11.40)	< 0.001	5.45(2.44–13.90)	< 0.001
CRF55_01B	36(3.10)	20(55.56)	1.43(0.72–2.83)	0.305	1.47(0.74–3.00)	0.157
B	35(3.01)	10(28.57)	0.46(0.21–0.98)	0.043	0.44(0.19–0.93)	0.037
CRF68_01B	24(2.07)	17(70.83)	2.78(1.13–6.83)	0.026	3.60(1.49–9.63)	0.006
Others^d^	39(3.36)	15(38.46)	0.72(0.37–1.40)	0.328	0.54(0.26–1.09)	0.093

^a^Numbers in parentheses indicate the constituent ratio of all the subjects in each variable

^b^Numbers in parentheses indicate the proportion of the clustered objects as a percentage of each subgroup

^c^Other includes IDU (*N* = 4, clustered = 3) and URF subtype (*N* = 9, clustered = 7)

^d^Other includes CRF08_BC subtype (*N* = 26, clustered = 13) and URF subtype (*N* = 13, clustered = 2)

**Fig. 1:**
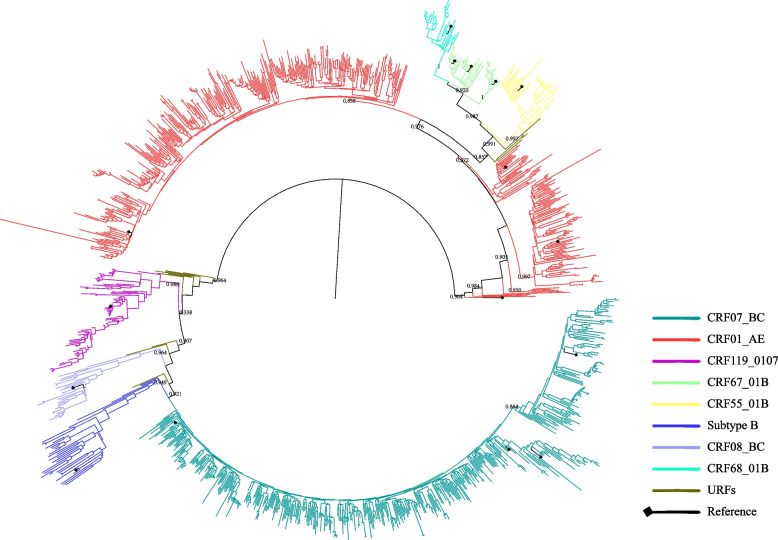
Phylogenetic tree analysis of nucleotide sequences from newly infected in Nanjing

Regarding HIV subtypes, the predominant subtype was CRF07_BC (40.57%, 471/1161), followed by CRF01_AE (38.41%, 446/1161), CRF119_0107 (6.29%, 73/1161), CRF67_01B (3.19%, 37/1161), CRF55_01B (3.10%, 36/1161), B (3.01%, 35/1161), CRF08_BC (2.24%, 26/1161), CRF68_01B (2.07%, 24/1161), and URFs (1.12%, 13/1161) (Fig. 1).

### Characteristics of HIV molecular transmission network

**Fig. 2:**
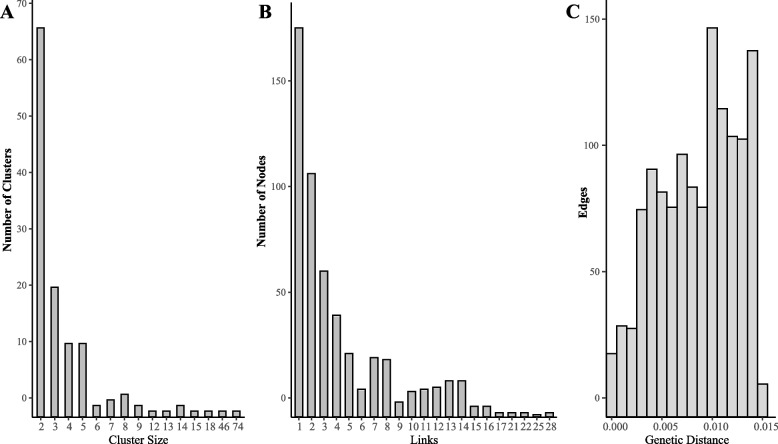
The characteristics of the molecular transmission networks. **A** Distribution of molecular transmission clusters by cluster size; **B** Distribution of nodes in clusters by links; **C** Distribution of edges by difference genetic distances

The TN93 model was utilized to calculate pairwise genetic distances under a 1.5% optimal genetic distance threshold. A total of 137 transmission clusters were identified, containing 613 individuals (52.80%) and 1381 edges, with cluster sizes ranging from 2 to 74 sequences (Fig. 2A). Among the 137 clusters, 69 clusters (50.36%) included 2 sequences, 49 (35.77%) clusters contained 3–5 sequences, 11 (8.03%) clusters contained 6–10 sequences, and 8 (5.84%) clusters consisted of 11 or more sequences. All nodes in the transmission network had the number of links ranging from 1 to 28, with 184 (30.02%, 184/613) having one link with another node, 349 (56.93%) having 2–10 links with other nodes, 73 (11.91%) having 11–20 links with other nodes, and only 7 (1.14%) nodes having more than 20 links with other nodes (Fig. 2B). Within the 1.5% genetic distance threshold, 818 (59.23%) edges were found with less than 0.010 genetic distance. In total, 1381 edges were identified below the 1.5% genetic distance threshold (Fig. 2C).

Regarding the subtype distribution of clusters, there were 208 nodes with CRF01_AE, forming 64 clusters. Among these clusters, 62 were small clusters consisting of 176 nodes. Similarly, 250 nodes with CRF07_BC formed 43 clusters, with 39 small clusters comprising 136 nodes. Additionally, 30 nodes with CRF67_01B formed 6 clusters, and there were 5 small clusters with 16 nodes. For the CRF119_0107, a total of 63 nodes formed 5 clusters, with 4 of them being small clusters involving 17 nodes. Other subtypes such as CRF67_01B, CRF55_01B, CRF08_BC, subtype B, and URF had 17 nodes forming 4 clusters, 20 nodes forming 5 clusters, 13 nodes forming 4 clusters, 10 nodes forming 5 clusters, and 2 nodes forming 1 cluster, respectively. All of these clusters were small clusters.

**Fig. 3:**
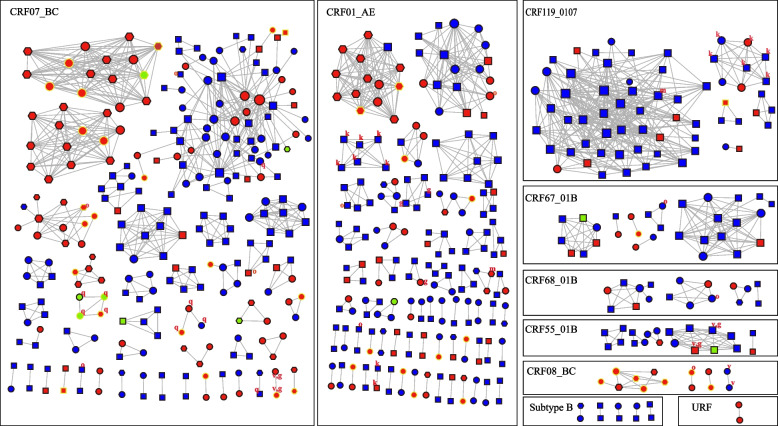
The molecular transmission networks of newly diagnosed individuals are depicted, with clusters categorized by subtype. Individuals aged clusters with subtype are shown. Age ≤ 30 years old are labeled with squares (■), those aged 31 ~ 59 years old are represented by circles (●), and individuals aged ≥ 60 years old are labeled with regular hexagons (  ). Patients infected through heterosexual contact are shown in red, homosexual contact in blue, and other types of contact in green. Male individuals are indicated by a black border, while female individuals are indicated by an orange border. Letters are used to highlight cases of TDR, with k, q, g, v, m and o indicating K103N/KN, Q58E/QE, G190A, V179D/E, M41ML and other TDR mutations, respectively. The size of the nodes indicates the number of edges connected to the node, and the more connections, the larger the node size

The largest molecular cluster was CRF07_BC, which consisted of 71 males and 3 females, with the primary mode of transmission being homosexual transmission (71.62%). A total of 28 clusters involved female individuals. Among the analyzed clusters, 23 transmission clusters contained 44 individuals infected with TDR strains. Of these clusters, 9 (39.13%) clusters were identified as having a shared transmission relationship among TDR individuals. Among the above 9 clusters, 6 clusters composed of all TDR individuals. As for TDR mutations in the transmission network, the main PI-associated mutations Q58E/QE were all distributed in CRF07_BC cluster, NNRTI-associated mutations K103N/KN in CRF01_AE and CRF119_0107 cluster, as well as V179D/E and G190A in CRF07_BC and CRF_5501B cluster, respectively (Fig. 3).

### Analysis of individuals with potential transmission links

Among the 1161 patients analyzed, there were 613 nodes within 137 clusters, accounting for 52.80% (613/1161). Univariate and multivariate logistic regression models were used to analyze the data, and the results were presented in Table 1. The multivariate analysis showed that compared with individuals aged ≤ 30 years old, those aged ≥ 60 years old were more likely to cluster (*OR* = 2.10, 95%*CI* = 1.18–3.79, *P* = 0.013). Employed patients were less likely to cluster than unemployed patients (*OR* = 0.67, 95%*CI* = 0.47–0.96, *P* = 0.029). Patients whose samples were obtained from sources other than VCT were more likely to cluster than those from VCT (*OR* = 1.69, 95%*CI* = 1.09–2.66, *P* = 0.021). Patients with CD4 cell counts between 200 ~ 500 or above 500 were more likely to cluster than those with CD4 cell counts below 200 (200 ~ 500: *OR* = 1.95, 95%*CI* = 1.20–3.21, *P* = 0.008; above 500: *OR* = 2.03, 95%*CI* = 1.19–3.50, *P* = 0.010). Regarding viral genealogies, subtype B was considerably less likely to cluster than CRF01_AE (*OR* = 0.44, 95%*CI* = 0.19–0.93, *P* = 0.037). Furthermore, CRF119_0107 (*OR* = 8.36, 95%*CI* = 4.32–17.90, *P* < 0.001), CRF67_01B (*OR* = 5.45, 95%*CI* = 2.44–13.90, *P* < 0.001), and CRF68_01B (*OR* = 3.60, 95%*CI* = 1.49–9.63, *P* = 0.006) were more likely to cluster than CRF01_AE (Table 1).

### Characterization of large clusters

In our study, eight large clusters (with more than 10 nodes) were identified, containing a total of 206 individuals (191 males and 15 females). Among these clusters, the major infection route was homosexual transmission (58.74%), followed by heterosexual transmission (40.29%), and other transmission routes (0.97%) (Table 2). A total of 5 TDR cases were distributed across 4 large clusters.

**Table 2:** Characteristics of the large molecular transmission clusters

Cluster No	Subtype	Nodes	Genetic distances Mean[Median, IQR]	Age	Gender	Transmission route	Drug resistance
1	CRF07_BC	74	0.012 [0.013, 0.011—0.014]	A1(35), A2(37), A3(2)	M(71), F(3)	Hetero(20), Homo(53), other(1)	Y(2), N(72)
2	CRF105_0107	46	0.0092 [0.010, 0.0066—0.012]	A1(41), A2(5)	M(46)	Hetero(5), Homo(41)	Y(1), N(45)
3	CRF01_AE	18	0.010 [0.011, 0.0086—0.012]	A1(9), A2(9)	M(18)	Hetero(6), Homo(12)	Y(1), N(17)
4	CRF07_BC	15	0.0054 [0.0057, 0.0038—0.0067]	A2(6), A3(9)	M(10), F(5)	Hetero(14), other(1)	N(15)
5	CRF01_AE	14	0.0078 [0.0086, 0.0047—0.011]	A1(3), A2(10), A3(1)	M(12), F(2)	Hetero(13), Homo(1)	N(14)
6	CRF67_01B	14	0.0083 [0.0095, 0.0057—0.011]	A1(11), A2(3)	M(14)	Hetero(1), Homo(13)	N(14)
7	CRF07_BC	13	0.0076 [0.0076, 0.0057—0.0095]	A2(4), A3(9)	M(11), F(2)	Hetero(13)	N(13)
8	CRF07_BC	12	0.011 [0.012, 0.011—0.013]	A2(6), A3(6)	M(9), F(3)	Hetero(11), Homo(1)	Y(1), N(11)

A1: age group1 (16–30 yrs), A2: age group2 (31–59 yrs), A3: age group3 (≥ 60 yrs)

*M* male, *F* female, *Hetero* heterosexual contact, *Homo* homosexual contact, *Y* TDR, *N* no TDR

In the large clusters, the proportion of patients in the ≥ 60 years old group was higher than that in the ≤ 30 years old group (*OR* = 4.14,95%*CI* = 2.02–8.55, *P* < 0.001). The proportion of patients without TDR was higher than that of patients with TDR (*OR* = 5.32,95%*CI* = 2.15–16.30, *P* = 0.001). Regarding different subtypes, the proportion of individuals infected with CRF01_AE was lower than those infected with CRF119_0107 (*OR* = 40.92, 95%*CI* = 21.08–79.42, *P* < 0.001), CRF07_BC (*OR* = 4.48,95%*CI* = 2.91–7.09, *P* < 0.001), and CRF67_01B (*OR* = 10.55,95%*CI* = 4.73–23.52, *P* = 0.002) (Table 3).

**Table 3:** Factors influencing the inclusion of individuals into the large molecular transmission clusters

Variables	Total N(%)^a^	Not included in a large cluster (≤ 10) N(%)^b^	Included in a large cluster (> 10) N(%)^b^	Univariate analysis		Multivariate analysis	
				*OR(95%CI)*	*P*	*aOR(95%CI)*	*P*
Gender							
Male	1090(93.88)	899(82.48)	191(17.52)				
Female	71(6.12)	56(78.87)	15(21.13)	1.26(0.70–2.28)	0.442		
National							
Others	24(2.07)	20(83.33)	4(16.67)				
Han	1137(97.93)	935(82.23)	202(17.77)	1.08(0.37–3.19)	0.889		
Marital status							
Married	336(28.94)	268(79.76)	68(20.24)				
Unmarried	725(62.45)	604(83.31)	121(16.69)	0.79(0.57–1.10)	0.161		
Divorced	100(8.61)	83(83.00)	17(17.00)	0.81(0.45–1.45)	0.474		
Age							
16 ~ 30	629(54.18)	532(84.58)	97(15.42)				
31 ~ 59	430(37.03)	359(83.49)	71(16.51)	1.08(0.78–1.51)	0.634	1.18(0.76–1.84)	0.459
≥ 60	102(8.79)	64(62.75)	38(37.25)	3.26(2.06–5.14)	< 0.001	4.14(2.02–8.55)	< 0.001
Occupation							
Unemployed	178(15.33)	148(83.15)	30(16.85)				
Students	187(16.11)	166(88.77)	21(11.23)	0.62(0.34–1.14)	0.124	0.50(0.24–1.03)	0.062
Employee	559(48.15)	464(83.01)	95(16.99)	1.01(0.64–1.58)	0.965	0.94(0.56–1.59)	0.807
Peasant	88(7.58)	53(60.23)	35(39.77)	3.26(1.82–5.82)	< 0.001	1.79(0.85–3.80)	0.124
Others	149(12.83)	124(83.22)	25(16.78)	0.99 (0.56–1.78)	0.986	0.49(0.24–0.98)	0.045
Education							
Low	256(22.05)	191(74.61)	65(25.39)				
High	905(77.95)	764(84.42)	141(15.58)	0.54(0.39–0.76)	< 0.001	0.81(0.49–1.35)	0.420
Sample source							
VCT	610(52.54)	522(85.57)	88(14.43)				
Hospital	443(38.16)	349(78.78)	94(21.22)	1.60(1.16–2.20)	0.004	0.98(0.65–1.49)	0.941
Others	108(9.30)	84(77.78)	24(22.22)	1.69(1.02–2.81)	0.041	1.56(0.83–2.86)	0.161
Disease classification							
AIDS	198(17.05)	169(85.35)	29(14.65)				
HIV	963(82.95)	786(81.62)	177(18.38)	1.31(0.86–2.01)	0.211		
Baseline HIV VL (log RNA copies/ml)							
≤ 3	57(4.91)	48(84.21)	9(15.79)				
> 3	1104(95.09)	907(82.16)	197(17.84)	1.16(0.56–2.40)	0.692		
Baseline CD4 cell count (cell/mm3)							
< 200	260(22.39)	218(83.85)	42(16.15)				
200 ~ 500	655(56.42)	530(80.92)	125(19.08)	1.22(0.83–1.80)	0.301		
> 500	246(21.19)	207(84.15)	39(15.85)	0.98(0.61–1.57)	0.927		
Transmission route							
Homosexual	800(68.91)	679(84.88)	121(15.13)				
Heterosexual	348(29.97)	265(76.15)	83(23.85)	1.76(1.28–2.40)	< 0.001	1.12(0.71–1.77)	0.631
Others	13(1.12)	11(84.62)	2(15.38)	1.02(0.22–4.66)	0.979	0.64(0.08–3.04)	0.610
STD history							
Yes	201(17.31)	174(86.57)	27(13.43)				
No	810(69.77)	655(80.86)	155(19.14)	1.53(0.98–2.37)	0.061		
Unknown	150(12.92)	126(84.00)	24(16.00)	1.23(0.68–2.23)	0.500		
TDR							
Yes	91(7.84)	86(94.51)	5(4.49)				
No	1070(92.16)	869(81.22)	201(18.79)	3.98(1.59–9.93)	0.004	5.32(2.15–16.30)	0.001
Subtype							
CRF01_AE	446(38.41)	414(92.83)	32(7.17)				
CRF07_BC	471(40.57)	357(75.80)	114(24.20)	4.13(2.72–6.27)	< 0.001	4.48(2.91–7.09)	< 0.001
CRF105_0107	73(6.29)	27(36.99)	46(63.01)	22.04(12.15–40.00)	< 0.001	40.92(21.08–79.42)	< 0.001
CRF67_01B	37(3.19)	23(62.16)	14(37.84)	7.88(3.70–16.76)	< 0.001	10.55(4.73–23.52)	< 0.001
Others	134(11.54)	134(100.00)	0(0.00)	-	-	-	-

^a^Numbers in parentheses indicate the percentage of subjects in each variable

^b^Numbers in parentheses indicate the percentage of clustered objects within each subgroup

## Discussion

Eight subtypes were identified in this study, which exceeded the six prevalent subtypes found in Shanghai and Hangzhou [16, 17]. This highlights the local HIV genetic diversity and the complexity of the HIV-1 epidemic among the population in Nanjing. Interestingly, contrary to previous studies [18], CRF07_BC has emerged as the main dominant strain, surpassing CRF01_AE. A meta-analysis has also suggested that CRF07_BC would become the dominant circulating strain among MSM [19]. The prevalence of CRF07_BC has surpassed that of CRF01_AE among MSM in Nanjing [20–22], indicating that the HIV epidemic in Nanjing was predominantly driven by homosexual transmission [9]. This may explain the increasing popularity of CRF07_BC. Similar trends have been observed in Shenzhen [12].

In our study, we discovered several novel circulating recombinant forms that have gained popularity in China in recent years, including CRF119_0107, CRF55_01B, CRF67_01B, and CRF68_01B [23, 24]. Particularly noteworthy was the emergence of HIV-1 second-generation recombinant strain composed of CRF01_AE and CRF07_BC, known as CRF119_0107, which has become the third most prevalent strain. This recombinant strain was first reported in the MSM population in Nanjing [25]. Previous studies by Wei Li have shown that CRF01_AE and CRF07_BC strains were already prevalent in Nanjing, with the earliest strains dating back to the 1980s-1990s [20, 21]. CRF01_AE and CRF07_BC were the main circulating strains among sexually transmitted populations, particularly in the MSM population [18, 26]. After nearly 30–40 years of evolution and transmission, some MSM populations became infected with both CRF01_AE and CRF07_BC, leading to the emergence of second-generation recombinant CRF119_0107 (CRF01_AE/CRF07_BC). Multiple second-generation recombinant forms (CRF01_AE/CRF07_BC) have been reported nationwide in recent years, such as CRF80_0107, CRF102_0107, CRF109_0107, and CRF123_0107 [27–30]. Additionally, certain second-generation recombinant forms (CRF01_AE/CRF07_BC), such as CRF79_0107 and CRF125_0107, have also been observed in heterosexual populations [31, 32]. Notably, we found that for the first time, a female individual was infected with the CRF119_0107 strain. This suggests that the CRF119_0107 subtype has started to spread from the MSM population to other populations. Considering the diversity and change trends of HIV-1 subtypes in Nanjing, it is necessary to conduct a comparative analysis with the national and global HIV-1 subtype background in the future, so as to carry out targeted surveillance, investigation and publicity interventions.

The risk of HIV transmission increases with higher access rates. In our study, more than half have entered the molecular network, which was higher than the access rates under the same gene distance threshold reported in Guangzhou (42.9%) [33] and Baoding (14.0%) [34]. This indicates a higher risk of local HIV transmission in Nanjing. In 2017, several MSM were infected with CRF119_0107 strains [25]. After several years of virus transmission, CRF119_0107 has formed a large transmission cluster with the highest access rate, which indicates that it has spread rapidly in Nanjing. Therefore, further surveillance work should be carried out.

The access rates of CRF67_01B and CRF68_01B were also high, with CRF67_01B forming a large cluster consistent with a previous study in Jiangsu. CRF67_01B mainly spreads within Jiangsu, with few reports from other provinces [19]. Further study found that CRF67_01B is growing rapidly in Wuxi City and is concentrated in young MSM populations. In this study, nearly four-fifths of the young MSM in the CRF67_01B transmission cluster were from Nanjing, which suggested that Nanjing was promoting the spread of the CRF67_01B epidemic. It has been reported that from 2015 to 2019, CRF67_01B and CRF68_01B experienced a rapid growth stage in 2014–2015 and then remained stable in Nanjing [24]. This finding also emphasizes the need for monitoring and intervention regarding the CRF67_01B and CRF68_01B subtypes to prevent the formation and spread of large clusters of the CRF68_01B subtype. In 2017–2018, the access rates of CRF01_AE and CRF07_BC in Jiangsu Province were 32.97% and 43.56%, respectively, which were lower than those in Nanjing. Further study found that Nanjing accounted for the highest proportion in the network [19]. CRF01_AE and CRF07_BC have the characteristics of interprovincial transmission in Hefei, with some cases related to Jiangsu Province [35]. As a neighboring city of Hefei and an important city in the Yangtze River Delta, Nanjing is more attractive. The frequent population communication in Nanjing and the long-term prevalence of CRF01_AE and CRF07_BC in Nanjing may lead to the trans-regional transmission of these strains.

We found that several factors influence access to HIV-1 molecular transmission networks, including age, occupation, sample source, and baseline CD4 cell count. Younger individuals, compared to older patients, tend to have higher mobility, and their infection sources may come from other places. A study on partner testing of HIV-infected individuals in Zhejiang showed that nearly 80% of the partners who were successfully tested positive for HIV were newly diagnosed infections [36]. Furthermore, research has shown that HIV patients diagnosed through passive detection have a higher prevalence of delayed HIV diagnosis [37]. In comparison to those diagnosed at voluntary counseling and testing (VCT) clinics, the lower rate of access to HIV-1 molecular transmission networks among HIV-infected individuals diagnosed through VCT may be related to earlier detection of their own diagnosis and delayed detection of the sexual partner who caused their infection. Previous studies have demonstrated that CD4 cell count continues to decrease in untreated HIV-infected populations [38, 39]. Due to the long duration of infection among individuals with initially low CD4 cell counts and population migration, it becomes difficult for them to enter the molecular transmission network.

Homosexual and bisexual individuals can contribute to the accelerated spread of HIV with higher efficiency [19]. In our study, the first three large clusters were composed of CRF07_BC, CRF119_0107, and CRF01_AE, respectively. These clusters included not only MSM but also heterosexual males, with MSM being the main component. This phenomenon can be explained by the fact that some heterosexual individuals were actually MSM or bisexuals, as previously reported [40]. We found that MSM were linked with heterosexual individuals in three-quarter large molecular clusters. This serves as a warning sign that we need to strengthen intervention efforts targeting MSM to halt the rapid spread of the epidemic and prevent the transmission of HIV from MSM to other populations. Although the access rate of CRF07_BC was higher than that of CRF01_AE, there was no statistical difference; however, CRF07_BC was more likely to form large clusters. We also observed that CRF07_BC had a higher proportion of patients aged 60 years and older, and these older individuals were more likely to be included in larger transmission clusters. Similar to the findings of a study conducted in Zhangjiajie [41], we found that older patients aged 60 years and above were more likely to acquire or transmit HIV within their own region. This can be attributed to the limited activities of the elderly within their local area and the common occurrence of unprotected non-marital sex among them [42], which further promotes the spread of HIV. Hence, we will carry out prospective continuous monitoring of molecular transmission networks, timely identify expanding molecular clusters and core sources of transmission, and seek key places in combination with in-depth interviews, so as to carry out targeted interventions to curb the HIV epidemic.

The overall prevalence of TDR in our study was 7.84%, which was significantly higher than the prevalence rate in Jiangsu (3.20%) [43] and nationwide (4.51%) [44]. The increased availability of ART for newly diagnosed HIV patients may lead to an increase in TDR [45]. Additionally, the prevalence of TDR was related to regional socioeconomic development to some extent [46]. For instance, high-income regions such as Shanghai and Tianjin have relatively high TDR prevalence rates, with rates of 17.4% and 12.2%, respectively [17, 47]. As the capital of Jiangsu Province, Nanjing has a relatively high economic level, and its high prevalence rate is therefore reasonable. It is worth noting that TDR has been transmitted in small clusters, highlighting the need for future TDR monitoring and individual intervention efforts.

NNRTI-associated mutation K103N/KN, G190A as well as V179D/E was detected most frequently in transmission network, which induced efavirenz (EFV) and nevirapine (NVP) resistance, consistent with previous research [48, 49]. In China, EFV or NVP has been used as a free first-line ART regimens since 2004. Due to the long-term use of these drugs, high prevalence of NNRTI-associated mutations associated with EFV and NVP resistance in ART patients, which further leads to the emergence of NNRTI-associated TDR. Similar to the Shenyang study [50], we found PI-associated mutation Q58E/QE was all detected in CRF07_BC cluster, which induced tipranavir/ritonavir (TPV/r) resistance. Fortunately, TPV/r was not included as a free antiviral drug and rarely used in China [51, 52], therefore, Q58E has little impact on the selection of ART prescriptions for the TDR patients. This means that it is necessary to implement optimized treatment regimens for cases resistant to first-line drugs in the network and continuously monitor molecular clusters with TDR cases, otherwise it will lead to reduced efficacy of ART and further spread of TDR. Furthermore, the TDR surveillance should be strengthened and the list of ART drugs should be expanded to facilitate the optimization of treatment regimen.

This study had some limitations. Frist, despite our efforts to collect samples from all newly diagnosed HIV patients, it was inevitable that some patients could not be included in the analysis due to untraceable personnel. Second, we classified 9 patients with unknown infection routes and 4 IDU patients into one category, namely others. This may cause some deviation.

## Conclusion

In this study, we conducted a systematic and comprehensive analysis of the molecular transmission network of among newly reported HIV cases in Nanjing from 2019 to 2021. Our findings suggested that the composition of HIV molecular subtypes was complex and diverse, with the high risk of local HIV transmission, the rapid spread of CRF119_0107 and the high TDR prevalence. Therefore, it is crucial to implement targeted interventions for the molecular transmission clusters identified in the study to effectively control the HIV epidemic.

### Supplementary Information

**Supplementary Material 1.**

## Data Availability

The gene sequences in this paper have been deposited in the GenBase in National Genomics Data Center, Beijing Institute of Genomics, Chinese Academy of Sciences/China National Center for Bioinformation, under accession number: C_AA055237.1—C_AA056397.1 that is publicly accessible at https://ngdc.cncb.ac.cn/genbase.

## References

[1] He N, Detels R (2005). The HIV epidemic in China: history, response, and challenge. Cell Res.

[2] Lyu P, Chen FF (2019). National HIV/AIDS epidemic estimation and interpretation in China. Zhonghua Liu Xing Bing Xue Za Zhi.

[3] Oster AM, France AM, Mermin J (2018). Molecular Epidemiology and the Transformation of HIV Prevention. JAMA.

[4] Fauci AS, Redfield RR, Sigounas G, Weahkee MD, Giroir BP (2019). Ending the HIV Epidemic: A Plan for the United States. JAMA.

[5] Guideline of monitor and intervention technique of HIV transmission network in China (trial version) has been issued. https://ncaids.chinacdc.cn/zxzx/zxzx/201909/t20190929_205904.htm.

[6] Oster AM, Wertheim JO, Hernandez AL, Ocfemia MC, Saduvala N, Hall HI (2015). Using Molecular HIV Surveillance Data to Understand Transmission Between Subpopulations in the United States. J Acquir Immune Defic Syndr.

[7] Han X, Zhao B, An M, Zhong P, Shang H (2020). Molecular network-based intervention brings us closer to ending the HIV pandemic. Front Med.

[8] Vrancken B, Zhao B, Li X, Han X, Liu H, Zhao J, Zhong P, Lin Y, Zai J, Liu M et al: Comparative Circulation Dynamics of the Five Main HIV Types in China. J Virol 2020;94(23). 10.1128/JVI.00683-20 PMC7654276 32938762

[9] Zhu Z, Xu Y, Wu S, Li X, Shi H, Dong X, Xu W (2022). Survival and risk factors associated with mortality in people living with HIV from 2005 to 2018 in Nanjing. China Front Public Health.

[10] Shi L, Tang W, Liu X, Hu H, Qiu T, Chen Y, Xu X, Chen Y, Zhang Z, Zhou Y (2022). Trends of late HIV presentation and advance HIV disease among newly diagnosed HIV cases in Jiangsu, China: A serial cross-sectional study from 2008 to 2020. Front Public Health.

[11] Xu JJ, Han MJ, Jiang YJ, Ding HB, Li X, Han XX, Lv F, Chen QF, Zhang ZN, Cui HL (2021). Prevention and control of HIV/AIDS in China: lessons from the past three decades. Chin Med J (Engl).

[12] Zhang D, Zheng C, Li H, Li H, Liu Y, Wang X, Jia L, Chen L, Yang Z, Gan Y (2021). Molecular surveillance of HIV-1 newly diagnosed infections in Shenzhen, China from 2011 to 2018. J Infect.

[13] Pang X, Tang K, He Q, Huang J, Fang N, Zhou X, Zhu Q, Wu X, Shen Z, Liang S (2021). HIV drug resistance and HIV transmission risk factors among newly diagnosed individuals in Southwest China. BMC Infect Dis.

[14] Kosakovsky Pond SL, Weaver S, Leigh Brown AJ, Wertheim JO (2018). HIV-TRACE (TRAnsmission Cluster Engine): a Tool for Large Scale Molecular Epidemiology of HIV-1 and Other Rapidly Evolving Pathogens. Mol Biol Evol.

[15] Li D, Chen H, Li H, Ma Y, Dong L, Dai J, Jin X, Yang M, Zeng Z, Sun P (2022). HIV-1 pretreatment drug resistance and genetic transmission network in the southwest border region of China. BMC Infect Dis.

[16] Zhang J, Guo Z, Yang J, Pan X, Jiang J, Ding X, Zhang W, Xia Y, Xu Y, Huang J (2015). Genetic diversity of HIV-1 and transmitted drug resistance among newly diagnosed individuals with HIV infection in Hangzhou. China J Med Virol.

[17] Wang Z, Zhang M, Zhang R, Liu L, Shen Y, Wang J, Lu H (2019). Diversity of HIV-1 genotypes and high prevalence of pretreatment drug resistance in newly diagnosed HIV-infected patients in Shanghai, China. BMC Infect Dis.

[18] Li W (2021). Analysis of molecular evolution and molecular network characteristics of HIV-1 in Nanjing.

[19] Yin Y (2021). Molecular and social transmission networks of MSM populations newly reported HIV-1 infection in Jiangsu province.

[20] Li W, Chu J, Wei F, He Y, Dong X, Ge Y, Ji Y, Musa TH, Cao S, Ni Q (2019). Molecular characteristic of HIV-1 CRF01_AE in Nanjing from 2015 to 2017. Infect Genet Evol.

[21] Li W, Li X, He Y, Ge Y, Ong JJ, Li X, Dong X, Chu J, Musa TH, Cao S, et al. The evolutionary and transmission characteristic of HIV-1 CRF07_BC in Nanjing, Jiangsu. J Med Virol. 2020;92(12):3237–45. 10.1002/jmv.25854 32275071

[22] Liu Y, Li R, Li X, Li W, Chu J, Wei P (2022). Molecular epidemiologic characteristics and transmission network of HIV-1 CRF01_AE strain in Nanjing. Chinese journal of AIDS & STD.

[23] Gan M, Zheng S, Hao J, Ruan Y, Liao L, Shao Y, Feng Y, Xing H (2021). The prevalence of CRF55_01B among HIV-1 strain and its connection with traffic development in China. Emerg Microbes Infect.

[24] Ge Y, Liu Y, Fu G, Lu J, Li X, Du G, Fei G, Wang Z, Li H, Li W (2022). The Molecular Epidemiological and Immunological Characteristics of HIV-1 CRF01_AE/B Recombinants in Nanjing. China Front Microbiol.

[25] Yin Y, Zhou Y, Lu J, Guo H, Chen J, Xuan Y, Yuan D, Hu H, Xu X, Fu G (2021). First Detection of a Cluster Novel HIV-1 Second-Generation Recombinant (CRF01_AE/CRF07_BC) among Men Who Have Sex with Men in Nanjing. Eastern China Intervirology.

[26] Li X, Li W, Zhong P, Fang K, Zhu K, Musa TH, Song Y, Du G, Gao R, Guo Y (2016). Nationwide Trends in Molecular Epidemiology of HIV-1 in China. AIDS Res Hum Retroviruses.

[27] Zhang Y, Pei Z, Li H, Han J, Li T, Li J, Liu Y, Li L (2019). Characterization of a Novel HIV-1 Circulating Recombinant Form (CRF80_0107) Among Men Who Have Sex with Men in China. AIDS Res Hum Retroviruses.

[28] Li X, Wu J, Zhang Y, Shen Y, Li H, Xing H, Liu Y, Yang X, Ding X, Hu B (2019). Characterization of a novel HIV-1 second-generation circulating recombinant form (CRF102_0107) among men who have sex with men in Anhui. China J Infect.

[29] Wang X, Zhao J, Li X, Li H, Zhang Y, Liu Y, Chen L, Zheng C, Jia L, Han J (2020). Identification of a novel HIV-1 second-generation Circulating Recombinant Form CRF109_0107 in China. J Infect.

[30] Xing Y, Wang L, Li Y, Wang Y, Han L, Huang G, Han J, Zhang W, Jia L, Liu Y (2022). Identification of a new HIV-1 intersubtype circulating recombinant form (CRF123_0107) in Hebei province. China J Infect.

[31] Li Y, Feng Y, Li F, Xue Z, Hu J, Xing H, Ruan Y, Shao Y (2017). Genome Sequence of a Novel HIV-1 Circulating Recombinant Form (CRF79_0107) Identified from Shanxi. China AIDS Res Hum Retroviruses.

[32] Xiao M, Feng Y, Gao L, Yang C, Liu J, He M, Li J, Zhang M, Dong X, Xia X (2022). Characterization of a Newly Emerging HIV-1 Second-Generation Recombinant Form (CRF125_0107) Among Heterosexuals in Yunnan. China J Infect.

[33] Yang J, Han Z, Xu H, Xing H, Xu P, Cheng W, Gu Y, Lyu F (2021). Identifying the Key Nodes of HIV Molecular Transmission Network Among Men Who Have Sex with Men - Guangzhou, Guangdong Province, China, 2015–2017. China CDC Wkly.

[34] Shi P, Chen Z, Meng J, Su M, Yang X, Fan W, Shi H, Gao Y, Lu X (2021). Molecular transmission networks and pre-treatment drug resistance among individuals with acute HIV-1 infection in Baoding, China. PLoS ONE.

[35] Zheng S, Wu J, Hu Z, Gan M, Liu L, Song C, Lei Y, Wang H, Liao L, Feng Y, et al. Epidemiology and Molecular Transmission Characteristics of HIV in the Capital City of Anhui Province in China. Pathogens. 2021;10(12):1554. 10.3390/pathogens10121554 PMC8708547 34959509

[36] Ni ZK, Luo MY, Pan XH, Jiang J, Chen L, Xia SC (2019). Related factors on sexual partners regarding receipt of HIV test among HIV positive men who have sex with men in Zhejiang province. Zhonghua Liu Xing Bing Xue Za Zhi.

[37] Chen J, Xu J, Zhou Y, Luo Y. HIV Detection and Delayed Diagnosis: A Time Series Analysis in China. Int J Environ Res Public Health. 2022;19(24). 10.3390/ijerph192416917 PMC9778823 36554798

[38] Fahey JL, Taylor JM, Detels R, Hofmann B, Melmed R, Nishanian P, Giorgi JV (1990). The prognostic value of cellular and serologic markers in infection with human immunodeficiency virus type 1. N Engl J Med.

[39] Goujard C, Bonarek M, Meyer L, Bonnet F, Chaix ML, Deveau C, Sinet M, Galimand J, Delfraissy JF, Venet A (2006). CD4 cell count and HIV DNA level are independent predictors of disease progression after primary HIV type 1 infection in untreated patients. Clin Infect Dis.

[40] Li X, Gao R, Zhu K, Wei F, Fang K, Li W, Song Y, Ge Y, Ji Y, Zhong P (2018). Genetic transmission networks reveal the transmission patterns of HIV-1 CRF01_AE in China. Sex Transm Infect.

[41] Wu YQ, Zou XB, Qin R, He JM, Zhang PF, Jiang Y, Chen GM, Yang YJ, Chen X (2016). Correlativity of subtype B viral transmission among elderly HIV-1 infected individuals in Yongding district, Zhangjiajie city, Hunan province. Zhonghua Liu Xing Bing Xue Za Zhi.

[42] Xu Y, Zhu Z, Wu S (2022). Analysis on characteristics of high-risk sexual behaviors before confirmation of HIV infections/AIDS patients aged50 years old in Nanjing City. Occup health.

[43] Guo H, Xu X, Hu H, Zhou Y, Yang H, Qiu T, Fu G, Huan X (2015). Low prevalence of the transmitted HIV-1 drug resistance among newly diagnosed HIV-1 individuals in Jiangsu Province, China during 2009–2011. BMC Public Health.

[44] Yuan H, Liu Z, Wu X, Wu M, Fang Q, Zhang X, Shi T, Tully DC, Zhang T (2021). Prevalence of transmitted HIV-1 drug resistance among treatment-naive individuals in China, 2000–2016. Arch Virol.

[45] Huang HY, Daar ES, Sax PE, Young B, Cook P, Benson P, Cohen C, Scribner A, Hu H (2008). The prevalence of transmitted antiretroviral drug resistance in treatment-naive patients and factors influencing first-line treatment regimen selection. HIV Med.

[46] Pennings PS (2013). HIV Drug Resistance: Problems and Perspectives. Infect Dis Rep.

[47] Zheng MN, Ning TL, Zhou N, Zhao X, Li L, Zhu JJ, Cheng SH (2018). Transmitted drug resistance among HIV infected men who have sex with men in Tianjin, 2014–2017. Zhonghua Liu Xing Bing Xue Za Zhi.

[48] Hong H, Tang C, Liu Y, Jiang H, Fang T, Xu G (2023). HIV-1 drug resistance and genetic transmission network among newly diagnosed people living with HIV/AIDS in Ningbo, China between 2018 and 2021. Virol J.

[49] Zuo L, Liu K, Liu H, Hu Y, Zhang Z, Qin J, Xu Q, Peng K, Jin X, Wang JH (2021). Corrigendum to "Trend of HIV-1 drug resistance in China: A systematic review and meta-analysis of data accumulated over 17 years (2001–2017)" [EClinicalMedicine 18 (2020) 100238]. EClinicalMedicine.

[50] Zhao B, Song W, Kang M, Dong X, Li X, Wang L, Liu J, Ding H, Chu Z, Wang L (2021). Molecular Network Analysis Reveals Transmission of HIV-1 Drug-Resistant Strains Among Newly Diagnosed HIV-1 Infections in a Moderately HIV Endemic City in China. Front Microbiol.

[51] Zhao B, Song W, An M, Dong X, Li X, Wang L, Liu J, Tian W, Wang Z, Ding H (2021). Priority Intervention Targets Identified Using an In-Depth Sampling HIV Molecular Network in a Non-Subtype B Epidemics Area. Front Cell Infect Microbiol.

[52] AIDS and Hepatitis C Professional Group, Society of Infectious Diseases, Chinese Medical Association (2018). Chinese Center for Disease Control and Prevention: [Chinese guidelines for diagnosis and treatment of HIV/AIDS (2018)]. Zhonghua Nei Ke Za Zhi.

