# Supplementary material for: Molecular transmission network analysis of newly diagnosed HIV-1 infections in Nanjing from 2019 to 2021
Source: BMC Infect Dis. 2024 Jun 12;24:583. doi: 10.1186/s12879-024-09337-6 (PMC11170874; doi:10.1186/s12879-024-09337-6)
Supplement: Supplementary file 1 — Supplementary Material 1. [file 12879_2024_9337_MOESM1_ESM.docx]

**Table S1. Primers for PCR and sequencing in the study**

| Procedure | Name | Location (HXB2) | Sequences (5$'$-3$'$) |
| --- | --- | --- | --- |
| The first round RT-PCR | F1a | 2057 - 2085 | TGAARGAITGYACTGARAGRCAGGCTAAT |
|  | F1b | 2068 - 2092 | ACTGARAGRCAGGCTAATTTTTTAG |
|  | RT-R1 | 3370 - 3348 | ATCCCTGCATAAATCTGACTTGC |
| The second round PCR | PRT-F2 | 2243 - 2266 | CTTTARCTTCCCTCARATCACTCT |
|  | RT-R2 | 3326 - 3304 | CTTCTGTATGTCATTGACAGTCC |
| Sequencing | SeqF3 | 2556 - 2577 | AGTCCTATTGARACTGTRCCAG |
|  | SeqR3 | 2639 - 2619 | TTTYTCTTCTGTCAATGGCCA |
|  | SeqF4 | 2869 - 2889 | CAGTACTGGATGTGGGRGAYG |
|  | SeqR4 | 2952 - 2931 | TACTAGGTATGGTAAATGCAGT |
